# Liver Metabolism at the Crossroads: The Reciprocal Control of Nutrient-Sensing Nuclear Receptors and Autophagy

**DOI:** 10.3390/ijms26125825

**Published:** 2025-06-18

**Authors:** Eun Young Kim, Jae Man Lee

**Affiliations:** 1 Department of Biochemistry and Cell Biology, Cell and Matrix Research Institute, School of Medicine, Kyungpook National University, 680 Gukchaebosang-ro, Daegu 41944, Republic of Korea; key11@knu.ac.kr; 2 BK21 FOUR KNU Convergence Educational Program of Biomedical Sciences for Creative Future Talents, Department of Biomedical Science, The Graduate School, Kyungpook National University, Daegu 41944, Republic of Korea

**Keywords:** nuclear receptor, PPARα, FXR, autophagy, liver

## Abstract

Peroxisome proliferator-activated receptor α (PPARα, encoded by NR1C1) and farnesoid X receptor (FXR, encoded by NR1H4) are the two prominent nutrient-sensing nuclear receptors essential for maintaining hepatic metabolism during fasting and fed states, respectively. These nuclear receptors comprehensively regulate the transcription of numerous genes involved in fatty acid oxidation (FAO), ketogenesis, bile acid (BA) biosynthesis, and other metabolic processes critical for liver energy homeostasis. These receptors have been shown to have opposite impacts on autophagy, which is triggered by PPARα activation but inhibited by FXR activation. Recent studies have further revealed that liver-specific genetic ablation of key autophagic genes tremendously impairs the activation of these nuclear receptors, thereby profoundly affecting hepatic metabolism in both fasting and feeding states. This review explores the roles and mechanisms of PPARα and FXR in regulating liver metabolism and autophagy, highlighting the necessity of basal autophagic activity in ensuring the proper signaling of these nutrient-sensing nuclear receptors. Finally, we examine the potential therapeutic strategies that leverage the interplay between PPARα, FXR, and autophagy for the treatment of metabolic liver disorders. We also delve into the clinical implications of this complex relationship, emphasizing its significance for translational medicine and future therapeutic interventions.

## 1. Introduction

The liver is a core metabolic organ for processing key nutrients, carbohydrates, fats, and proteins from diets, synthesizing and distributing glucose, lipids, and ketone bodies to extrahepatic tissues, and converting excess nitrogen to urea. Feeding and fasting transitions allow the liver to turn from carbohydrate-enriched energy deposition in the fed state to FAO-mediated energy consumption in the fasted state [1]. A substantial number of transcription factors, including nuclear receptors, play key roles in these metabolic transitions. Among 48 members of the nuclear receptor superfamily, there are two distinctive nutrient-sensing nuclear receptors: PPARα (also known as NR1C1) and FXR (also known as NR1H4).

PPARα is activated in the fasted state of the liver and is known as the master transcription factor of hepatic FAO and ketogenesis. Its activation in the fasted state also stimulates gluconeogenesis [2]. On the other hand, it is strongly believed that FXR is activated in the fed state in response to several species of BAs returning to the liver, together with intestinally absorbed fat-soluble nutrients, through enterohepatic circulation. FXR is essential for BA homeostasis as a BA sensor in the nucleus. It also has significant impacts on the suppression of other metabolic processes, such as gluconeogenesis and lipogenesis [3].

Besides the reciprocal antagonism of these receptors on the regulation of gluconeogenesis, the expression of these two nutrient-sensing nuclear receptors is regulated by each other. FXR transactivation, in response to treatment with natural or synthetic FXR agonists, such as chenodeoxycholic acid (CDCA) and GW4064, increases the expression of human *PPARα* but not mouse *Pparα* [4]. PPARα, on the other hand, is necessary for boosting the expression of *Fxr* in the fasted mouse liver [5]. As a result, each nutrient condition is likely to prepare the other by increasing the expression of the appropriate nutrient sensor.

Consistent with these opposing but complementary roles, these nuclear receptors have been found to govern autophagy, another adaptive response in the liver with significant metabolic implications [6]. Conversely, accumulating studies suggest that hepatic autophagy activity markedly affects PPARα or FXR-dependent metabolic pathways such as FAO, ketogenesis, and BA homeostasis [7,8,9,10,11]. Ablation of core autophagy genes in a hepatocyte-specific manner remarkably blunts the expression of target genes of PPARα, FXR, or liver X receptor α (LXRα, also known as NR1H3), which are involved in FAO, ketogenesis, BA biosynthesis, and de novo lipogenesis, leading to the development of intrahepatic cholestatic injury. Earlier studies have also supported this by showing that nuclear accumulations of nuclear receptor corepressor 1 (NCoR), one of the well-defined corepressors, inhibit the transactivation of PPARα, FXR, or LXRα in these mutant animals [7,8,12]. Recent findings, including our studies, suggest another potential mechanism by which the activities of nuclear receptors are compromised in autophagy-deficient hepatocytes. Enhanced cytoplasmic interactions between p62 (also known as sequestosome 1) and kelch-like ECH-associated protein 1 (KEAP1) in the liver of autophagy-defective mutant mice promote the translocation of nuclear factor erythroid 2-related factor 2 (NRF2), a key transcription factor for antioxidant defense, from the cytoplasm to the nucleus. This process appears to hinder the transactivation of PPARα and FXR, although the underlying mechanisms remain unclear [9,10,11].

In this review, we summarize recognized overlapping and interdependent metabolic roles of PPARα and FXR in liver energy homeostasis. We discuss how these receptors transcriptionally coordinate hepatic autophagy and how basal autophagy activity reciprocally impacts the functional roles of these two nutrient sensors. Finally, we explore potential therapeutic strategies to harness the functional interactions of these receptors with autophagy to treat various liver disorders.

## 2. Nutrient-Sensing Nuclear Receptors PPARα and FXR

The 48 members of the human nuclear receptor superfamily function as transcriptional switches that translate environmental and metabolic signals into genomic responses, modulating gene expression via ligand-dependent or independent mechanisms [13,14]. These receptors orchestrate almost every aspect of mammalian physiology, and their dysfunctions often lead to diverse human disorders, including liver diseases [15]. They are important therapeutic targets for pharmacological interventions, with research focusing on tissue-specific or time-restricted approaches. Some nuclear receptor-targeting drugs have been approved and are widely used to treat inflammatory and metabolic diseases, such as inflammation, hyperglycemia, hyperlipidemia, and hypertension [16,17,18]. Among these, PPARα and FXR stand out as prominent nutrient-sensing nuclear receptors, coordinating metabolic adaptation to changes in nutritional status, such as fasting and feeding, respectively. By regulating lipid, glucose, and bile acid metabolism, they play crucial roles as both physiological regulators and therapeutic targets for metabolic diseases [2,19].

### 2.1. Fasting-Activated Nuclear Receptor PPARα

PPARα is a ligand-dependent transcription factor that belongs to the NR1C subfamily of the nuclear receptor superfamily, along with PPARβ/δ (NR1C2) and PPARγ (NR1C3). All PPAR isoforms form heterodimers with retinoid x receptors (RXRs) and bind to peroxisome proliferator response elements (PPREs) located in the regulatory regions of target genes. These PPREs are typically composed of two AGGTCA hexamer sequences arranged as a direct repeat separated by a single nucleotide (DR-1) [20,21,22,23].

PPARα plays a critical role in the fasting state by transcriptionally activating genes involved in fatty acid uptake, FAO, ketogenesis, and lipid metabolism, particularly in the liver. Its biological relevance is highlighted by its clinical use as a therapeutic target of fibrate-class drugs for treating hyperlipidemia in humans [24].

**Figure 1:**
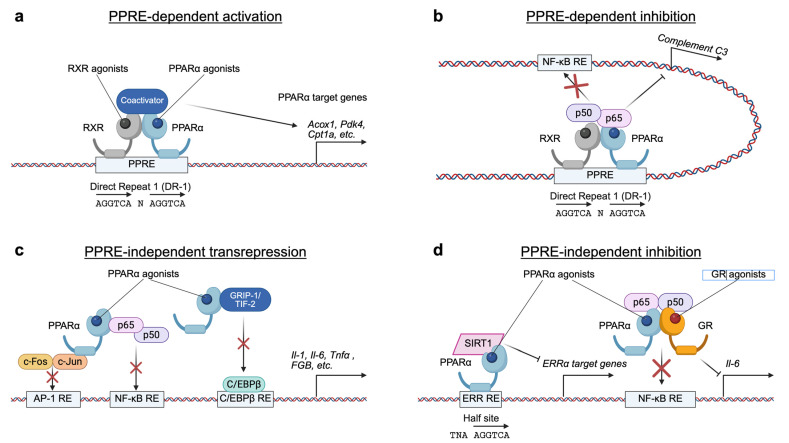
Various mechanisms of transcriptional regulation by PPARα. (**a**) PPRE-dependent activation. In the presence of specific agonist ligands, the PPARα-RXR heterodimer complex binds to PPREs in the regulatory regions (e.g., promoters or enhancers) of target genes and recruits coactivator complexes, leading to the expression of genes involved in lipid and glucose metabolism. (**b**) PPRE-dependent inhibition. Upon binding to agonist ligands, agonist-bound PPARα-RXR on the PPRE physically interacts with p65 and interferes with its activity, antagonizing its binding to an NF-kB response element (RE) in the complement *C3* promoter. (**c**) PPRE-independent transrepression. PPARα directly interacts with several transcription factors and coregulators such as AP-1 (c-Fos-C-Jun), NF-kB (p65-p50), and GRIP-1/TIF-2, preventing their binding to cognate response elements and suppressing target gene expression. (**d**) PPRE-independent inhibition. PPARα binds to ERR RE and recruits to SIRT1, thereby inhibiting ERRα target genes. The ligand-activated PPARα-GR heterodimer complex inhibits TNF-induced IL-6 expression via a mechanism involving a physical interaction with NF-kB. RE, response element; N, any nucleotide.

PPARα plays a crucial role in regulating energy metabolism, particularly in highly oxidative tissues such as the liver, skeletal muscle, and heart. As a master transcription factor, PPARα orchestrates the expression of numerous genes involved in lipid and glucose metabolism, with a well-defined role in the process of FAO [25,26,27,28,29,30,31]. During periods of fasting, PPARα becomes activated and functions as a potent transactivator, upregulating the expression of key enzymes and proteins involved in fatty acid transport, triglyceride (TG) hydrolysis, FAO, and ketogenesis [32,33]. This coordinated regulation of gene expression by PPARα ensures the efficient utilization of fatty acids (FAs) as an energy source during energy deprivation. The importance of PPARα in this process is further demonstrated by studies on *Pparα*-null (*Pparα^−/−^)* mice, which show profound metabolic abnormalities when fasted, including hepatic steatosis, hypoglycemia, and hypoketonemia [25,26]. These phenotypes were attributed to the lack of robust FAO induction by PPARα activation in both peroxisomes and mitochondria [25,26,34]. In addition to acting as a transactivator, PPARα activation can downregulate the expression of genes associated with inflammation, complement, and coagulation, which is achieved by its physical interaction and subsequent interference with the activity of pro-inflammatory transcription factors, such as nuclear factor-κB (NF-κB), activating protein-1 (AP-1), and signal transducer and activator of transcription (STAT) [35,36,37]. PPARα activation by fibrates also intercepts the coactivator glucocorticoid receptor-interacting protein-1 (GRIP-1)/transcriptional intermediary factor-2 (TIF-2) of CCAAT box/enhancer-binding protein β (C/EBPβ), downregulating IL-6-mediated expression of the gene encoding fibrinogen β [38]. A PPARα-SIRT1 complex represses the expression of ERRα target genes involved in mitochondrial respiration via a direct binding to a single hexameric ERR response element [39,40]. Simultaneous activation of PPARα and glucocorticoid receptor (GR) synergistically transrepresses NF-kB-driven gene expression [41]. Thus, PPARα can act as both a transactivator and transrepressor, depending on the presence of PPREs and/or other interacting transcription factors of the target genes [42] (Figure 1).

PPARα expression is dynamically regulated by various physiological and metabolic cues: conditions that increase its expression include fasting, hormones (e.g., growth hormone, leptin, and glucocorticoids), and circadian rhythm; conditions that suppress PPARα expression include insulin, inflammatory cytokines (e.g., TNFα, IL-1β, IL-6), metabolites (e.g., glucose, glucose-1-phosphate, glucose-6-phosphate), and aging [26,43,44,45,46,47,48,49,50,51,52,53,54,55,56,57,58,59,60,61,62,63]. In addition to the autoregulation of its expression, the levels and activity of the PPARα are also affected by other transcription factors such as Kruppel-like factor 6 (KLF6), hepatocyte nuclear factor 4 (HNF4), chick ovalbumin upstream promoter-transcription factor II (COUP-TFII), liver X receptor (LXR), and pregnane X receptor (PXR) [4,64,65,66,67]. Moreover, it has been shown that other NRs forming heterodimers with RXR can compete with PPARα, resulting in the inhibition of PPARα activity in cells or tissues where RXR is limited [68]. Ligand availability and specificity also regulate the stability and turnover of PPARα [65,69].

Meanwhile, substantial effort has been devoted to identifying the endogenous ligands for PPARα. These include a variety of FAs and their derivatives: long-chain polyunsaturated FAs (e.g., linoleic acid), acyl-CoAs, oxidized FAs (e.g., phytanic acid), phospholipids (e.g., phosphatidylcholine 16:0-18:1), eicosanoids (e.g., 8S-HETE, leukotriene B_4_), and endocannabinoids (e.g., oleoylethanolamide) [42,70,71,72,73,74,75,76,77,78,79,80,81]. In particular, it is highly appealing to consider the possibility that the increased levels of free FAs could activate PPARα in the fasted liver, providing these nutrients with an extra function by serving as agonist ligands to promote their consumption. This idea is further supported by the discovery that hepatic PPARα activation during fasting requires adipose triglyceride lipase (ATGL)-dependent lipolysis in white adipose tissues. Adipocyte-specific *Atgl* knockout mice were shown to profoundly impair ketone body production and fibroblast growth factor 21 (FGF21) secretion in the fasted state of the liver [82]. It is of interest to note that the liver of wild-type mice fed a high-fat, low-carbohydrate ketogenic diet (KD) showed a marked increase in the expression of PPARα target genes, including *Fgf21*, although dietary supplementation of essential fatty acids has not been demonstrated to activate PPARα [83,84]. In other studies, “new” hepatic fats derived from de novo lipogenesis (DNL) mediated by fatty acid synthase (FAS) have been suggested as a necessary biochemical process for PPARα activation [85,86]. It has also been suggested that the endogenous agonist bound to the liver PPARα is a relatively abundant phosphatidylcholine (PC) species (PC 16:0-18:1) based on direct biochemical analysis using lipid extraction followed by electrospray ionization mass spectrometry [81]. Moreover, oleoylethanolamide, one species of endocannabinoids synthesized in the intestine, has also been proposed as a putative endogenous agonist with significant satiety effects [87]. Despite all these studies, the precise identity of endogenous ligands for PPARα remains uncertain.

Historically, PPARα was first identified for its propensity to stimulate peroxisome proliferation in rodents exposed to hypolipidemic agents such as Wy-14,643 [88]. Long-term pharmacological activation of PPARα by Wy-14,643 in mice leads to marked peroxisome proliferation, hepatocyte hyperplasia, and a high incidence of hepatocellular carcinoma (HCC) in over 70% of wild-type mice. However, this carcinogenic response appears to be species-specific. In humans, chronic treatment with PPARα agonists, such as gemfibrozil and fenofibrate, does not induce peroxisome proliferation or HCC and instead provides lipid-lowering benefits with a favorable safety profile in hyperlipidemic patients [89,90,91]. This interspecies difference underscores the importance of contextual and translational caution in extrapolating rodent findings to human clinical outcomes, particularly regarding PPARα-targeted therapies and liver cancer risk.

The function of PPARα is also extensively regulated by diverse coregulator recruitments [42]. In the absence of agonist ligands, PPARα binds corepressors such as NCoR, silencing mediator for retinoid or thyroid-hormone receptors (SMRT), and/or the receptor-interacting protein 140 (RIP140) [92,93,94]. These corepressors inhibit PPARα transactivation by competing with coactivators and recruiting histone deacetylases (HDACs). Upon binding to agonist ligands, a conformational change of the last helix corresponding to the AF-2 motif of the LBD expels the corepressor complex but recruits coactivators such as PPARγ coactivator 1α/β (PGC-1α/β), CREB binding protein (CBP)/p300, steroid receptor coactivator-1 (SRC-1), PPARα-interacting factor (PRIC), and/or mediator complex subunit 1 (MED1) [95,96,97]. Some of these coactivators have intrinsic histone acetyltransferase activity, facilitating chromatin remodeling [98,99,100]. The recruitment of coregulator complexes fine-tunes PPARα activity in response to various cellular signals and metabolic states.

As with other NRs, PPARα function is also regulated by various posttranslational modifications (PTMs), including phosphorylation, ubiquitination, and SUMOylation [69,101,102]. These PTMs lead to diverse outcomes of PPARα activity by affecting its stability, susceptibility to proteasomal degradation, recruitment of coregulators, and transactivation capability. The effects of these PTMs also depend on the specific residues modified and the stimuli and enzymes involved. In particular, PPARα turns out to be a phosphoprotein, and its activity is affected by several kinases, including mitogen-activated protein kinases (MAPKs: e.g., ERK1/2, c-Jun N-terminal kinases (JNK), p38), AMP-dependent protein kinase (AMPK), protein kinase A (PKA), protein kinase C (PKC), and glycogen synthase kinase 3β (GSK3β) [44,102,103,104,105,106,107,108,109,110].

The mammalian/mechanistic target of rapamycin complex 1 (mTORC1), another important nutrient sensor, significantly impacts hepatic PPARα activation. It has been demonstrated that PPARα activation in the fed state of the liver can be decreased by phosphorylation and its subsequent nuclear translocation of NCoR executed by mTORC1 and its downstream substrate kinase ribosomal protein S6 kinase B2 (S6K2) [111,112]. However, other studies have revealed that the constitutive activation of mTORC1 by hepatocyte-specific *Tsc1* deletion is insufficient to inhibit PPARα-mediated FAO and ketogenesis in the fasted state [113,114]. Hepatic PPARα activation can also be increased by the kinase-independent coactivation of AMPKα subunit, an intracellular energy sensor activated by a high AMP-to-ATP ratio in the fasted state [115]. Glucose supplementation represses expression of PPARα target genes via inactivation of AMPK in pancreatic β-cells, although it is unclear whether a similar mechanism also exists in the liver [116,117]. Restoring hepatic expression levels of adiponectin receptors AdipoR1 and AdipoR2 in *db/db* mice enhances AMPK activity and PPARα signaling pathways, which reduce gluconeogenesis but increase FAO. This implies a certain endocrine signaling pathway from adipocytes to hepatocytes, comprised of adiponectin, AdipoRs, AMPK, and PPARα [118].

### 2.2. BAs-Activated Nuclear Receptor FXR

FXR was discovered in 1995 through two different approaches. In yeast two-hybrid screens using the ligand-binding domain of human RXRα as bait, two splicing variants of RXR-interacting protein 14 and 15 (RIP14 and RIP15) were first identified [119]. The same gene was also cloned using degenerative PCR primers corresponding to the highly conserved DNA-binding domain of nuclear receptors in the rat cDNA library. It was named an FXR based on its mild activation in response to supraphysiological concentrations of farnesoid [120]. While mice possess two FXR genes (*Nr1h4*/*FXRα* and *Nr1h5*/*FXRβ*), humans retain only functional FXRα (*NR1H4*), as FXRβ is a pseudogene. FXRα exists as four isoforms (FXRα1 to FXRα4) in humans and mice due to differential promoter usage and alternative splicing [121]. All FXRα isoforms form obligate heterodimers with RXRs to bind to farnesoid X response elements (FXREs) throughout the genome. The most common motifs of FXREs are inverted repeat-1 (IR-1) (inverted AGGTCA hexameric repeat spaced by one nucleotide) sequences [120,122]. Notably, FXRα2 and FXRα4 uniquely bind to everted repeat-2 (ER-2, everted AGGTCA hexameric repeat spaced by two nucleotides) DNA motifs independently of RXR [121,122]. These isoforms regulate genes with overlapping IR-1/ER-2 DNA motifs, which are critical for lipid metabolism and ammonia detoxification [121,123].

FXR is a versatile ligand-dependent nuclear receptor that functions as both a transcriptional activator and repressor, critical for systemic metabolic regulation. Expressed in the liver, intestine, adrenal gland, and kidney, FXR governs diverse physiological processes, including lipid and glucose metabolism, amino acid degradation and ureagenesis, steroid biosynthesis, and water balance [119,120,124,125,126,127,128]. A cornerstone of its function lies in maintaining BA homeostasis by tightly controlling target genes involved in enterohepatic circulation and hepatic BA synthesis [129]. In hepatocytes, FXR activation induces key genes encoding the orphan nuclear receptor small heterodimer partner (SHP/NR0B2), bile salt export pump (BSEP/ABCB11), phospholipid transfer protein (PLTP), the multidrug resistance proteins MDR3 and MRP2, while repressing sodium taurocholate cotransport peptide (NTCP/SLC10A1) and organic anion-transporting polypeptide 2 (OATP2) via SHP-dependent mechanisms. In enterocytes, FXR activation upregulates several target genes encoding ileal BA-binding protein (IBABP), fibroblast growth factor 15/19 (FGF15/19), organic solute transporter α/β (OSTα/β), but suppresses the apical sodium-dependent BA transporter (ASBT) expression [126]. This dual regulation reduces BA retention in enterocytes, promotes BA reabsorption into circulation, and enhances enterohepatic recycling, thereby preventing BA toxicity while optimizing metabolic adaptation to nutrient availability [130].

The discovery that BAs can bind to FXR as endogenous ligands has markedly expanded our understanding of their physiological roles. FXR is now widely recognized as an intracellular BA sensor in metabolic tissues. This finding has substantially transformed our view of BAs from mere digestive detergents for solubilizing and absorbing lipophilic nutrients in the small intestine to important endocrine hormones regulating BA metabolism [131]. A couple of BA species can activate FXR in some way, and the potency of major BA activation is ordered as follows: CDCA > deoxycholic acid (DCA) > lithocholic acid (LCA) > cholic acid (CA) [132,133,134]. On the other hand, several species of BAs can also function as FXR antagonists. For example, tauro-β-muricholic acid (TβMCA) inhibits intestinal FXR activation in mice, but no equivalent BA species has been identified in humans [135,136]. However, a recent study using untargeted metabolomics in mouse tissues has uncovered that BA-methylcysteamine (BA-MYC) conjugates act as intestinal FXR antagonists, which reduce hepatic lipid accumulations in mouse models of hypercholesterolaemia. Unlike TβMCA, BA-MYCs were also found in human serum [137]. It is of interest to note that ursodeoxycholic acid (UDCA), a C7 epimer of CDCA, has been suggested as an FXR antagonist in non-alcoholic fatty liver disease (NAFLD) patients; this lowers FGF19 levels while inducing cholesterol 7α-hydroxylase (CYP7A1), a rate-limiting enzyme of BA biosynthesis [138].

In addition to endogenous ligands, FXR modulation has been explored through both natural and synthetic ligands, offering diverse therapeutic opportunities [126]. Guggulsterones (GCs), plant-derived FXR antagonists, decrease hepatic cholesterol levels in mice fed with high-cholesterol diets and may inhibit SARS-CoV-2 infection by downregulating the angiotensin-converting enzyme 2 (ACE2) expression in the gastrointestinal and respiratory systems [139,140]. GW4046, a synthetic FXR agonist, improves hepatic steatosis and insulin resistance in diet-induced obese (DIO) or *ob/ob* mice, though poor bioavailability limits its usage in clinical trials [141,142,143]. A non-BA synthetic compound, Fexaramine, an intestine-restricted FXR agonist, induces ileal *Fgf15* (mouse ortholog of the human *FGF19* gene), promoting body weight loss, reducing inflammation and hepatic glucose output, and enhancing thermogenesis and white adipose tissues (WAT) browning without systemic FXR activation [144,145]. These advances culminated in the development of obeticholic acid (OCA/INT-747), a semi-synthetic 6α-ethyl-CDCA analog, approved for primary biliary cholangitis (PBC), a hepatic autoimmune disease leading to inflammation and destruction of the bile ducts [146,147,148,149,150]. However, OCA’s accelerated FDA approval for nonalcoholic steatohepatitis (NASH) patients was denied primarily due to its limited efficacy and potential side effects [151]. Together, these developments highlight the promise of tissue-specific FXR modulation for metabolic and liver disorders while underscoring the need for improved drug profiles and targeted therapeutic strategies.

**Figure 2:**
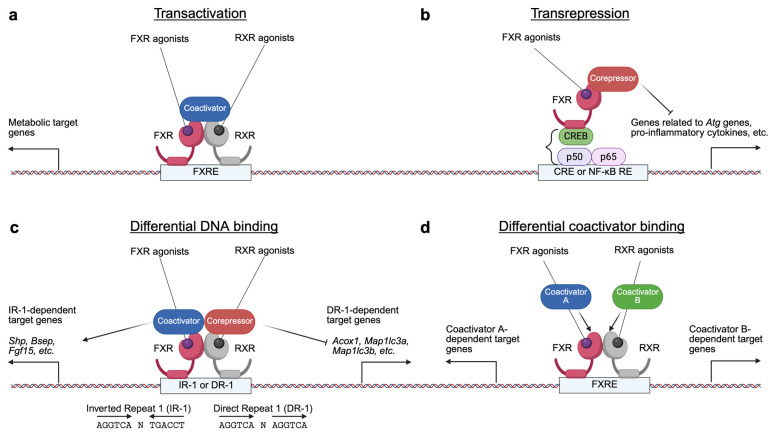
FXR-mediated transcription programs. (**a**) FXRE-dependent transactivation. In the presence of specific agonist ligands, the FXR-RXR heterodimer complex binds to FXREs in the regulatory regions (e.g., promoters or enhancers) of target genes and recruits coactivator complexes, leading to the expression of genes involved in BA and other metabolic pathways. (**b**) FXRE-independent transrepression. Upon agonist ligand binding, the FXR-RXR can physically interact with transcription factors such as CREB or NF-kB (p50-p65) and interfere with their activities, thereby antagonizing their binding to response elements in genes encoding autophagy-related proteins and pro-inflammatory cytokines. (**c**) Differential DNA binding. The FXR-RXR heterodimer can bind to different DNA motifs (e.g., IR-1 or DR-1), resulting in distinct transcriptional outcomes. (**d**) Differential coactivator binding. The recruitments of different coactivator complexes lead to the activation of differential target genes, reflecting the versatility of FXR-mediated transcriptional regulation. FXRE, farnesoid X response element; BA, bile acids; CRE, cAMP response element; RE, response element; N, any nucleotide.

FXR regulates gene expression through several transcriptional mechanisms (Figure 2). Upon activation by specific agonist ligands, the FXR-RXR heterodimer binds to FXREs in the promoters or enhancers of target genes, dissociating corepressor complexes but recruiting coactivator complexes to induce the expression of genes involved in BA and metabolic pathways. FXR can also mediate transrepression independently of FXREs by physical interactions with other transcription factors such as CREB or NF-kB, thereby inhibiting their activities and suppressing genes related to autophagy and inflammation. Additionally, the FXR-RXR complex can recognize different DNA motifs, including IR-1 and DR-1, resulting in opposite transcriptional outputs. The recruitment of various coactivator complexes further contributes to the specificity of FXR target gene regulation. Through these mechanisms, FXR plays a crucial role in maintaining metabolic homeostasis [152].

FXR, a nuclear BA receptor, is essential for maintaining BA homeostasis in the liver and gut [3]. Whole-body *Fxr* knockout (*Fxr^−/−^*) mice exhibit dysregulated hepatic BA biosynthesis, enlarged BA pools, and increased susceptibility to BA overload (e.g., severe cholestasis with wasting, hypothermia with reduced fat mass, and mortality on 1% cholic acid diets), reflecting impaired feedback regulation [129,153]. These mice also develop hepatic steatosis, steatohepatitis, fibrosis, and HCC, underscoring FXR’s role in metabolic health [154,155,156]. In addition to its role in BA homeostasis, mouse studies have suggested that FXR may support liver regeneration, mediate the metabolic benefits of bariatric surgery (e.g., vertical sleeve gastrectomy), and exhibit agonist-driven antitumor effects in colorectal cancers (CRC) [157,158,159]. Hepatic FXR activation is enhanced postprandially via O-GlcNAcylation of its AF1 domain, stabilizing the receptor and promoting transactivation by displacing corepressor complexes [160]. Furthermore, intestinal FXR activation stimulates the expression of the *Fgf15* gene and its subsequent secretion, which signals to the liver to enhance glycogen and protein synthesis and suppress BA synthesis [131,161]. Collectively, FXR integrates nutrient sensing, BA dynamics, and metabolic adaptation, marking its activation as a hallmark of metabolic equilibrium in the nourished liver.

## 3. Antagonistic Functions of PPARα and FXR in Hepatic Metabolism

**Figure 3:**
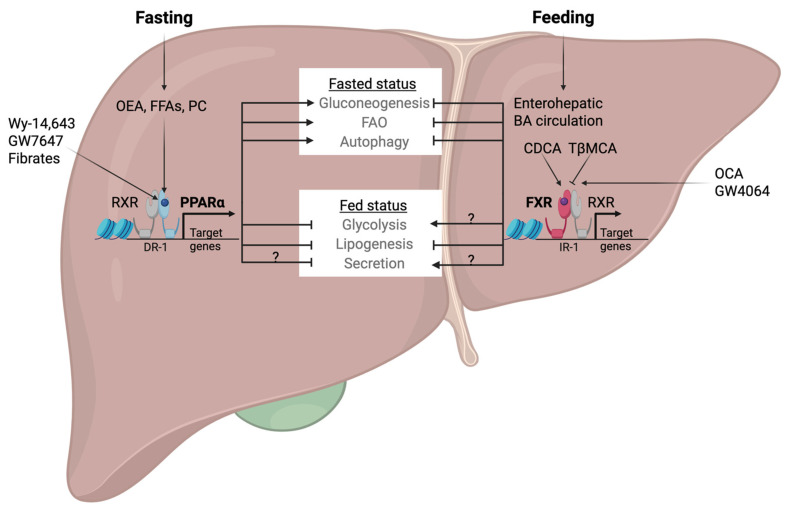
Coordination of hepatic nutrient metabolisms by PPARα- or FXR-mediated transcriptional programs. The effect of PPARα and FXR on secretion and the role of FXR on glycolysis remain unclear. Arrows indicate activation, while bars indicate repression. OEA, oleoylethanolamide; FFAs, free fatty acids; PC, phosphatidylcholine. Modified from [166,167].

The opposing responses of PPARα and FXR in the fasted or fed state of hepatic nutrient conditions imply that they might have the opposite effects on carbohydrate and fat metabolism and overall energy homeostasis in the liver (Figure 3).

Gluconeogenesis, for example, is an important biochemical pathway for energy balance mechanisms intimately associated with fasting and feeding cycles. Studies on *Pparα^−/−^* mice have shown severe fasting hypoglycemia, indicating the essential role of PPARα in maintaining fasting blood glucose levels at appropriate ranges [26]. Consistent with this, PPARα has been shown to induce the expression of several gluconeogenic genes [2,162]. However, stable isotope experiments have revealed that *Pparα^−/−^* mice produce more hepatic glucose, indicating increased glucose utilization in peripheral tissues such as skeletal muscle and adipose tissues. This highlights non-negligible discrepancies between gene expression patterns and real metabolic fluxes. Further experiments using hepatocyte-specific *Pparα* knockout mice may help to clarify these inconsistencies [163].

In contrast to PPARα, FXR activation reduces gluconeogenic gene expression by inducing the *Nr0b2* gene encoding SHP, the well-established nuclear receptor corepressor [124,142,164]. Nevertheless, some studies have reported that FXR activation can increase gluconeogenesis. Treatment of FXR agonists has been shown to stimulate the expression of hepatic phosphoenolpyruvate carboxylase (PEPCK), a rate-limiting enzyme of gluconeogenesis in various experimental models, including rat hepatoma cells, primary rat or human hepatocytes, and mice [165]. While there is context-dependent variability, PPARα and FXR tend to exhibit opposing metabolic roles in hepatic gluconeogenesis.

While PPARα and FXR often have opposing effects on liver metabolism, their roles in glucose utilization in the fed state show surprising similarities. Pharmacological activation of PPARα in response to fenofibrate or Wy-14,643 decreases glycolytic flux by inducing the gene encoding pyruvate dehydrogenase kinase 4 (PDK4), which inhibits the pyruvate dehydrogenase complex (PDC) in mouse and human hepatocytes, and by downregulating glycolytic genes encoding glucokinase and pyruvate kinase [31,168,169]. These inhibitory functions of PPARα in glycolysis are consistent with its physiological activation in the fasted state of the liver. Unexpectedly, FXR also suppresses glycolysis. *Fxr^−/−^* mice exhibit accelerated induction of glycolytic genes upon high carbohydrate refeeding, whereas the treatment of a synthetic FXR agonist in primary hepatocytes impairs glucose-mediated induction of the *Pklr* gene encoding liver-type pyruvate kinase (LPK). Mechanistically, activated FXR prevents carbohydrate response element-binding protein (ChREBP) from binding to carbohydrate response elements (ChORE) present in the regulatory regions of the *Pklr* gene [170]. Additionally, pharmacological FXR activation promotes *Pdk4* expression in mouse liver, rat hepatoma cells, and human primary hepatocytes [171]. Thus, both nuclear receptors appear to inhibit glycolysis in the fed state of the liver, revealing an unexpected convergence in their metabolic effects and suggesting a complex interplay between nutrient sensing and glucose metabolism.

Glycogen metabolism is another key energy balance pathway during feeding-fasting transitions. *Pparα^−/−^* mice exhibit lower quantities of hepatic glycogen, which might contribute to rapid hypoglycemia during the early hours of fasting [26,163,172]. It has been reported that the expression and activity of hepatic glycogen synthase, a rate-limiting enzyme of glycogenesis, rise in concert with the onset of glycogenolysis in the early phase of fasting, while PPARα activity begins to increase [173]. This process may be necessary for priming glycogenesis so that depleted glycogen stores can be replenished as soon as dietary glucose becomes available [174]. Consistent with this idea, pharmacological PPARα activation directly induces the *Gys2* gene encoding glycogen synthase 2 (GYS2) in rodent primary hepatocytes via its intronic DR-1 response element, whereas *Pparα^−/−^* mice show remarkably lower *Gys2* expression during refeeding after a prolonged fast [174]. However, synthetic PPARα agonists (e.g., fenofibrate, ciprofibrate) reduce hepatic glycogen and glucose-6-phosphate (G6P) levels in mice, although the underlying molecular mechanisms remain unclear [168,175]. Similarly, *Fxr^−/−^* mice also showed diminished fed-state hepatic glycogen content despite normal expression of genes encoding GYS2 and glycogen phosphorylase (GYG) [170,176]. In concordance with its roles in the fed state, GW4046-mediated FXR activation in *db/db* mice enhances hepatic glycogen synthesis by phosphorylating and inhibiting GSK3β, a kinase that normally suppresses glycogenesis [164]. This effect may involve the intestine-liver axis consisting of the intestinal FXR-FGF15/19 and hepatic fibroblast growth factor receptor 4 (FGFR4)-beta klotho (KLB) complex, suggesting cross-tissue coordination in glycogen regulation [161]. The opposing effects of PPARα (fasting-induced glycogen priming vs. agonist-driven depletion) and FXR (fed-state glycogen modulation) highlight context-dependent roles in hepatic energy storage.

FAO serves as a clear example of the opposing roles of PPARα and FXR in hepatic nutrient sensing. PPARα, a master regulator of fasting-induced metabolism, upregulates genes involved in mitochondrial and peroxisomal β-oxidation, as demonstrated by studies using PPARα agonists (e.g., Wy-14,643, GW7647, fenofibrate, etc.) and *Pparα^−/−^* mice [2,177]. Conversely, FXR activation appears to suppress FAO: bile acid-enriched diets impair PPARα transactivation by limiting coactivator recruitment, and *Fxr^−/−^* mice exhibit increased FAO gene expression [124,178]. Moreover, FXR activation by GW4064 reduces serum ketone levels in leptin receptor-deficient *db/db* mice, further suggesting FXR’s role in inhibiting hepatic FAO and ketogenesis [164]. These findings underscore FAO as another outstanding illustration where PPARα and FXR act antagonistically, balancing energy substrate utilization between fasting and fed states.

In line with its role in the fasting state, pharmacological activation of PPARα using fenofibrate suppresses hepatic lipogenesis by downregulating lipogenic genes, as observed in LDL receptor knockout (*Ldlr^−/−^*) mice on high-fat/high-cholesterol diets, fructose diets fed hamsters, and diabetes patients [179,180,181]. However, chronic fenofibrate administration in mice paradoxically induces both FAO and lipogenesis, through the sterol regulatory element-binding protein 1c (SREBP1c)-dependent activation of lipogenic pathways. This effect is not typically seen during physiological fasting [168]. FXR, on the other hand, suppresses hepatic lipogenesis primarily via the FXR-SHP axis, downregulating the *SREBF1* gene encoding SREBP1c and its downstream targets, such as fatty acid synthase (FASN) and acetyl-CoA carboxylase 1 (ACC1) [124,164,170,182]. Although some discrepancies remain regarding lipogenic response in the fed state, the overall effect of FXR appears to support lipid-lowering and metabolic normalization [183].

Taken together, PPARα and FXR represent key transcriptional regulators of liver metabolism during fasting and feeding cycles. While they often exert opposing effects, particularly in pathways such as FAO, gluconeogenesis, and glycogen metabolism, they may also converge in suppressing lipogenesis via overlapping regulatory mechanisms. This duality reflects the complexity of hepatic nuclear receptor signaling and highlights the importance of context-dependent regulation. A deeper understanding of this interplay is essential for the development of targeted therapies aimed at restoring metabolic homeostasis.

## 4. Opposing Transcriptional Coordination of Liver Autophagy by PPARα and FXR

Autophagy is an evolutionarily conserved intracellular catabolic process in all eukaryotic organisms, ranging from yeast to mammals, and serves as an essential mechanism for degrading and recycling cellular components [184,185]. This process delivers cytoplasmic constituents to lysosomes, where they undergo breakdown and subsequent recycling. Initially, autophagy was regarded as a non-selective degradation process of macromolecules, including carbohydrates, lipids, nucleic acids, and long-lived proteins [186]. However, recent studies have unveiled a more detailed understanding, revealing the existence of various forms of selective autophagy, targeting and degrading specific cellular components, including obsolete or damaged organelles such as mitochondria (mitophagy), peroxisomes (pexophagy), lysosomes (lysophagy), endoplasmic reticulum (ER-phagy), Golgi apparatus, ribosomes (ribophagy), portions of the nucleus (nucleophagy), bacteria (xenophagy), ferritin (ferritinophagy), glycogen (glycophagy), and lipid droplets (lipophagy). This selective targeting ensures efficient cellular quality control, host defense, and homeostasis maintenance [187,188]. Thus, autophagy plays a vital role in nutrient recycling during nutrient deprivation or fasting, allowing cells to meet their energy requirements by breaking down and reusing their components [189]. This adaptive mechanism not only supports cellular survival under stress conditions but also contributes to overall organismal health by removing potentially harmful cellular debris and maintaining metabolic balance [190,191].

**Figure 4:**
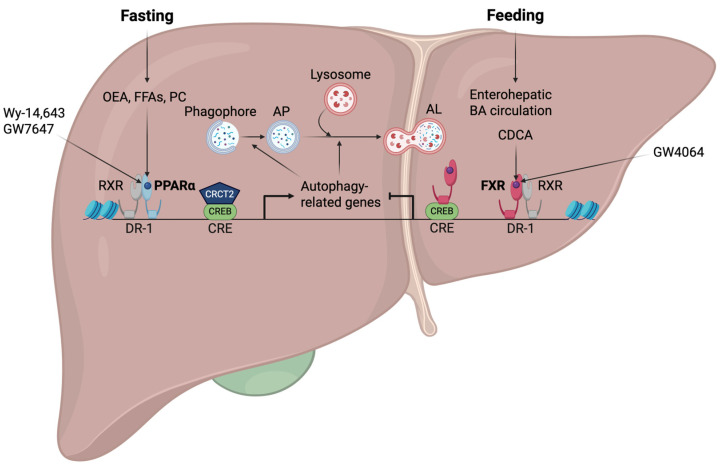
Transcriptional regulation of hepatic autophagy by PPARα, FXR, and CREB. PPARα activation by either fasting-induced endogenous ligands or pharmacological ligands induces many autophagy-related genes by binding to the DR-1 motif, together with RXR. The fasting-activated transcription factor CREB recruits the coactivator CRCT2 to increase the expression of autophagy-related genes. In contrast, FXR activation by either CDCA or GW4064 represses numerous autophagy-related genes by binding to the DR-1 motif together with RXR. FXR activation also dissociates and expels CRCT2 from the nucleus and forms a piggyback interaction with CREB, thereby downregulating autophagy-related genes. Arrows indicate activation, while bars indicate repression. CRCT2, CREB-regulated transcription coactivator 2. Modified from [6,192].

We investigated whether PPARα and FXR would have opposing impacts on the regulation of hepatic autophagy by considering their roles as nutritional sensors of fed and fasting situations. To explore this issue, fed or fasted wild-type, *Pparα^−/−^*, and *Fxr^−/−^* mice were orally administered the synthetic PPARα and FXR agonist ligands, GW7467 and GW4064, respectively [6]. Even though it was anticipated that PPARα would turn off in the fed state of the liver, autophagy was nevertheless promoted by PPARα activation in response to GW7647. Conversely, autophagy was suppressed by FXR activation by GW4064 treatment, even if it was expected to turn on in the fasted state of the liver. These two agonist ligands had contrasting effects on the transcriptional regulation of numerous autophagy-related genes in the livers of wild-type mice: GW7647 induced autophagy-related genes, but GW4046 suppressed them (Figure 4).

Next, to investigate whether these autophagy-related genes are direct targets of the two receptors, we conducted PPARα chromatin immunoprecipitation followed by next-generation sequencing (ChIP-seq) experiments to obtain precise information on the entire PPARα-binding sites throughout the whole genome (PPARα cistromes). Upon establishing PPARα cistromes from the livers of wild-type and *Pparα^−/−^* mice treated with either vehicle or GW764, they were compared with the previously established FXR cistromes in the Guo laboratory [122]. Our cistromic analysis not only revealed that significant binding peaks of PPARα and FXR were observed in the regulatory regions of many autophagy-related genes but also uncovered that those genes were substantially enriched among the major targets of both receptors [6].

Moreover, it has been demonstrated that the livers of *Pparα^−/−^* mice exhibited a marked reduction of autophagic vesicles during fasting, suggesting that PPARα is required for the physiological induction of autophagy. On the other hand, the anticipated reduction of autophagy in the fed state was likewise reversed in the liver of *Fxr^−/−^* mice, indicating that FXR is also required for the physiological suppression of autophagy during feeding [6]. Thus, these pharmacological interventions and genetically engineered mouse models highlight the physiological significance of both receptors as nutrient sensors in controlling autophagy. Another group has demonstrated that fasting increases the expression of major autophagy-related genes by recruiting the CREB-CRCT2 complex to their promoters. They have also confirmed that pharmacological FXR activation inhibits fasting-induced autophagy by disrupting the CREB-CRCT2 complex [192]. The *Rubcn* gene encoding Rubicon has been reported to be a novel FXR target gene in mouse livers. FXR activation upon OCA treatment induces hepatic *Rubcn* expression, which contributes to autophagy impairment by blocking the fusion process of APs and lysosomes [193].

Mechanistic analysis further demonstrated that PPARα and FXR could compete with each other to bind to DR-1 sites in the regulatory regions of autophagy-related genes, such as genes encoding microtubule-associated protein 1 light chain 3α and -β (LC3a and LC3b). FXR was not predicted to bind to such sites, although it was reported to repress the expression of genes encoding APOC III and APOA proteins via binding to DR-1 sites. This was verified by ChIP-qPCR analysis performed in the livers of wild-type mice treated with either vehicle or GW4046 in the fed and fasted state [194,195]. In accordance with these transcriptional repression mechanisms, GW4064-mediated FXR activation recruited corepressors such as NCoR and SMRT, resulting in increased repression marks of trimethylations on lysine 27 residue of histone 3 (H3K27me3). Based on these findings, we have proposed a working model in which PPARα and FXR can actively compete for binding to the promoters of the genes encoding LC3a and LC3b. Each agonist enhances the binding of its corresponding receptor while decreasing the binding of the other. Therefore, the binding competition between PPARα and FXR on the regulatory regions of autophagy-related genes results in opposing transcriptional output [6].

## 5. Phenotypic Abnormalities in Liver-Specific Knockout Mice of Core Autophagy-Related Genes

The molecular players of autophagy have been primarily discovered in genetic screen studies on the yeast *Saccharomyces cerevisiae*, which has led to the identification of over 30 autophagy-related (ATG) genes [196,197,198,199,200]. More than half of them are known to be core ATG genes necessary for AP formation. Surprisingly, these genes are highly conserved from yeasts to mammals, with a few mammalian-specific ones [197,201,202]. Dysfunctions of ATG genes are strongly associated with various human disorders, including metabolic and inflammatory diseases, infection, cancer, neurodegeneration, and aging [198,203,204,205,206]. Their significance in mammalian physiology was further demonstrated by investigating phenotypes of germline or conditional knockout mice. ATG genes are involved in each stage of the autophagy process, including autophagy initiation, vesicle nucleation, vesicle elongation, and AP-lysosome fusion. Although extensive studies have been conducted in various cell types, this section will focus on the metabolic pathogenicity arising from macroautophagy defects in mouse hepatocytes.

Autophagy initiation (the ULK1 complex): In mammals, autophagy initiation is coordinated by the unc-51-like kinase 1 (ULK1, a mammalian homology of yeast Atg1) complex, which integrates nutrient signals via mTORC1, AMP-activated protein kinase (AMPK), and others. Under nutrient-rich conditions, mTORC1 phosphorylates ULK1 and ATG13, suppressing autophagy. During starvation, mTORC1 is inactivated, allowing ULK1 activation and initiation of phagophore formation. AMPK further promotes this process under glucose deprivation. The ULK1 complex includes ULK1, FIP200 (also known as ATG11/RB1CC1), ATG14, ATG101, and ATG17, all components essential for its function [207].

Due to functional redundancy among different isoforms of ULKs, Yu et al. generated hepatocyte-specific *Ulk1* knockout mice on *Ulk2^−/−^* background (*Alb-Cre*; *Ulk1^F/F^*; *Ulk2^−/−^*) to assess hepatic ULK1/2 function. These mice showed normal autophagy despite mild hepatomegaly, with intact p62 turnover, LC3 lipidation, and AP formation, suggesting compensatory mechanisms. They were also protected from acetaminophen (APAP)-induced liver injury, likely due to reduced JNK pathway activation, and had unaltered hepatic lipid levels [208] (Table 1).

In contrast, hepatocyte-specific knockout (*Alb-Cre*; *Fip200^F/F^*) mice displayed severe hepatomegaly, p62 accumulation, and reduced TG levels in serum and liver during fasting or a high-fat diet (HFD) feeding. These changes were linked to impaired liver x receptor α (LXRα) signaling and greater susceptibility to endotoxin-induced liver damage, underscoring FIP200’s dual role in lipid metabolism and cytoprotection, beyond its canonical autophagy function [209]. No hepatocyte-specific knockout models of *Atg13*, *Atg17*, or *Atg101* have been reported to date (Table 1).

Vesicle nucleation (the Class III PI3K complex I): Vesicle nucleation at the phagophore is driven by the generation of phosphatidylinositol 3-phosphate (PI3P), catalyzed by the Class III phosphatidylinositol-3 kinase complex I (PI3KC3-CI). This complex includes vacuolar protein sorting 34 and 15 (Vps34 and Vps15, the catalytic and regulatory subunits of PI3K-CI, respectively), along with scaffolding proteins such as Beclin 1/Atg6, general vesicular transport factor p115, ATG14-like (ATG14L), an activating molecule in Beclin 1-regulated autophagy protein 1 (AMBRA1), and nuclear receptor-binding factor 2 (NRBF2). The activation of PI3KC3-CI is closely linked to the ULK1 initiation complex, which phosphorylates ATG14L, Vps34, and Beclin 1, facilitating the complex’s translocation to the phagophore assembly site (PAS) at the endoplasmic reticulum (ER)-derived omegasome. Local PI3P production recruits downstream effectors like WD repeat domain phosphoinositide-interacting protein 2 (WIPI2) and zinc-finger FYVE domain-containing protein 1 (DFCP1), promoting phagophore expansion and AP formation [207].

Liver-specific knockout studies have revealed key roles for PI3KC3-CI components in hepatic autophagy and metabolism. Hepatocyte-specific deletion of Vps34 (*Alb-Cre*; *Vps34^F/F^*) results in hepatomegaly, steatosis, and impaired AP formation, with reduced protein turnover and diminished amino acid-mediated mTOR signaling [210] (Table 2). Similarly, both acute and chronic liver-specific deletions of the *Vps15* gene cause liver enlargement, autophagy defects, p62 accumulation, and altered LC3 lipidation [211]. Chronic hepatocyte-specific *Vps15* knockout (*Alb-Cre*; *Vps15^F/F^*) mice display mitochondrial depletion, reduced FAO and ketogenesis, partly due to impaired PPARα activity from nuclear accumulation of corepressors NCoR and HDAC3 [7]. *Nrbf2* germline null mice also show reduced ATG14L-linked VPS34 activity, vesicle nucleation defects, and enhanced ER stress-mediated cytotoxicity, with focal liver necrosis and bile ductular hyperplasia [212]. To date, liver-specific knockouts of *Ambra1*, *Atg14l*, *Becn-1*, and *p115* have not been reported. These findings emphasize the essential role of PI3KC3-CI in maintaining hepatic autophagy and lipid metabolism, while underscoring the need for further research on its components in autophagy regulation and liver pathophysiology (Table 2).

Vesicle elongation (two ubiquitin-like conjugation systems): Vesicle elongation during autophagy is regulated by two ubiquitin-like conjugation systems: ATG12-ATG5 and ATG8-phosphatidylethanolamine (PE). The ATG12-ATG5 conjugate, formed by E1- and E2-like enzymes (ATG7 and ATG10), interacts with ATG16L1 to form a complex that localizes to the phagophore via WIPI2 [207]. ATG2 facilitates phospholipid delivery from the ER, while ATG9 helps redistribute lipids, promoting membrane expansion. Additional membrane contributions come from the plasma membrane, mitochondria, endosomes, and the Golgi complex [213,214,215].

The ATG8-PE system begins with the protease ATG4-mediated cleavage of mammalian homologs of yeast ATG8, such as microtubule-associated protein 1 light chain 3 alpha or beta (LC3A or LC3B) and the GABARAP subfamily (GABARAP, GABARAPL1, and GABARAPL2), exposing a C-terminal glycine. This form of LC3 protein (LC3-I) is conjugated with PE via the enzymatic actions of the ATG7, the E2-like enzyme ATG3, and the ATG12 complex, generating PE-conjugated LC3 protein (LC3-II), which anchors to the phagophore membrane and mediates cargo recruitment and AP closure. ATG4 recycles LC3-II by delipidation for subsequent conjugation cycles. These systems collectively ensure precise AP biogenesis and cargo encapsulation [207,213,214,215].

ATG3, essential for LC3 lipidation, is upregulated in patients and mice with nonalcoholic fatty liver disease (NAFLD) and promotes lipid accumulation. Knockdown of ATG3 reduces hepatic steatosis by enhancing FAO via JNK1 inhibition, increasing the activities of mitochondria and SIRT1. Thus, ATG3 influences both autophagy and lipid metabolism and may be a therapeutic target for NAFLD [216] (Table 3).

ATG5, a core component of the ATG12-ATG5-ATG16L1 complex, is essential for catalyzing LC3 lipidation and AP formation. In mosaic *Atg5*-deficient (*CAG-Cre*; *Atg5^F/F^*) mice, impaired proteostasis led to ubiquitin-positive aggregates, mitochondrial damage, oxidative stress, and benign liver adenomas [217,218]. Inducible *Mx1-Cre*; *Atg5^F/F^* mice showed progressive accumulation of ubiquitin proteins before large aggregate formation, indicating that early autophagy failure precedes visible pathology [219,228]. Chronic hepatocyte-specific *Atg5* knockout (*Alb-Cre*; *Atg5^F/F^*) mice exhibit apoptosis, inflammation, fibrosis, and HCC, which were attenuated by co-deletion of the gene encoding NRF2, suggesting adverse effects of NRF2 in autophagy-deficient liver injuries [220]. These mice also showed altered lipid metabolism, including reduced lipid accumulation and ketogenesis during fasting, highlighting ATG5’s role in starvation-induced lipid droplet (LD) formation and degradation [221,222].

ATG7, required for both conjugation systems, was studied in inducible *Mx1-Cre*; *Atg7^F/F^* mice, where hepatocyte deletion induced hepatomegaly, disorganized hepatic lobules, swollen and vacuolated hepatic cells with cell death, increased peroxisome biogenesis, deformed mitochondria, and elevated levels of ubiquitin-positive aggregates. Liver damage markers such as alanine aminotransferase (ALT), aspartate aminotransferase (AST), and alkaline phosphatase (ALP) were also increased, indicating severe liver injury [223]. *Alb-Cre*; *Atg7^F/F^* mice displayed conflicting phenotypes regarding lipid metabolism. Some studies reported hepatic steatosis with increased TG and cholesterol levels due to impaired LD degradation, while others observed opposite phenotypes during fasting [12,224,225,226]. Additional findings revealed improved glucose homeostasis via activating transcription factor 4 (ATF4)-FGF21 signaling, and impaired ketogenesis due to reduced PPARα activity [8,226]. Moreover, Inducible hepatocyte-specific *Atg7* knockout (*ERt-Alb-Cre*; *Atg7^F/F^*) mice challenged with D-galactosamine (GalN) and lipopolysaccharide (LPS) showed enhanced liver injury via caspase-9 and mitochondrial apoptosis, indicating a protective role of autophagy against inflammatory stress [227]. Despite variability in lipid phenotypes, all models consistently showed that ATG7 deficiency disrupts proteostasis, mitochondrial function, and redox balance, leading to liver degeneration and inflammation.

In summary, ATG5 and ATG7 play critical roles in maintaining hepatic homeostasis through autophagy, redox regulation, and metabolism. Their loss triggers liver dysfunction under both metabolic and inflammatory conditions, making them promising targets for treating liver diseases associated with autophagy impairment. Liver-specific knockouts of *Atg2*, *Atg4*, *Atg9*, *Atg10*, *Atg12*, *Atg16L1*, and mammalian homolog genes of yeast *Atg8* (LC3 proteins and GABARAP family proteins) remain unexplored and warrant further investigation (Table 3).

Docking and fusion of AP-lysosome (the Class III PI3K complex II): The maturation and fusion of APs with lysosomes require the class III PI3K complex II (PI3KC3-CII), which includes VPS34, VPS15, Beclin 1, and ultraviolet radiation resistance-associated gene protein (UVRAG). This complex also supports endosomal trafficking. During fusion, Pacer binds to syntaxin-17 (STX17) on AP and recruits the homotypic fusion and vacuole protein sorting (HOPS) complex, facilitating membrane tethering. UVRAG enhances AP-lysosome docking through its interaction with HOPS, while STX17 promotes membrane fusion for lysosomal degradation. Conversely, Rubicon (encoded by the *Rubcn* gene) inhibits autolysosome (AL) formation by binding to UVRAG and suppressing PI3KC3-CII activity. Small GTPases like the Ras-related protein Rab7a also regulate the fusion process by directing vesicle transport [229,230].

Hepatocyte-specific *Pacer* knockout (*Alb-Cre*; *Pacer^F/F^*) mice show defective autophagy flux, with TG and glycogen accumulation, reduced ketogenesis, and early-onset liver fibrosis, highlighting Pacer’s role in maintaining metabolic homeostasis [229] (Table 4). Conversely, hepatocyte-specific *Rubcn* knockout (*Alb-Cre*; *Rubcn^F/F^*) mice exhibit enhanced autophagy, protecting against HFD-induced hepatic steatosis, liver damage, and ER stress, although these mice appear normal on standard chow, indicating Rubicon’s stress-specific regulatory function [230]. To date, liver-specific knockout models of *Becn1*, *Uvrag*, and *Stx17* have not been reported (Table 4). Underscoring the need for further studies into their hepatic roles. These findings support the therapeutic potential of targeting AP-lysosome fusion machinery in metabolic liver disease.

## 6. Altered Nutrient-Sensing Nuclear Receptor Signaling in Liver-Specific Knockout Mice of Core-Autophagy Genes

Autophagy plays a pivotal role in controlling hepatic lipid metabolism, primarily via its effects on several nuclear receptor activities. Early studies in *Mx1-Cre*; *Atg7^F/F^* mice with inducible *Atg7* deletion revealed a global downregulation of genes involved in lipid metabolism, suggesting that autophagy is critical for sustaining the expression of both anabolic and catabolic lipid metabolism genes [231]. This finding underscores the groundwork for understanding autophagy’s broad impact on hepatic metabolic processes.

Further investigations using various autophagy-deficient mouse models provided more detailed insights. Studies using *Alb-Cre*; *Fip200^F/F^* mice demonstrated that impaired activation of nuclear receptors, particularly LXRα, resulted in reduced hepatic fat accumulation and increased susceptibility to liver injury from gut-derived endotoxins [209]. Similarly, *Alb-Cre*; *Atg7^F/F^* mice showed lower serum ketone bodies after 24-h fasting, indicating that autophagy deficiency alters lipid metabolism and ketogenesis. This study also showed a reciprocal relationship between PPARα and autophagy: PPARα upregulates genes encoding autophagy machinery and its regulatory proteins, while autophagy, in turn, influences PPARα activity [6].

Mechanistic insights into how autophagy deficits affect nuclear receptor activation emerged from the *Ulk1* knockdown study in the murine hepatocyte-derived AML12 cell line. In autophagy-defective hepatocytes, increased activity of ribosomal protein S6 kinase B1 (RPS6KB1, also known as S6K1) leads to the nuclear accumulation of the transcriptional corepressor NCoR, inhibiting LXRα activation. This results in reduced expression of the lipogenic *Scd1* gene and increased vulnerability to lipotoxicity [232]. These findings parallel previous reports in mice where genetic activation of mTORC1 or its downstream effector S6K2 increased NCoR phosphorylation and its nuclear translocation, inactivating the fasting-activated nuclear receptor PPARα and reducing FAO and ketogenesis [111,112]. Despite the compelling evidence linking autophagy to nuclear receptor regulation via NCoR, some studies have presented conflicting results. While genetic activation of hepatic mTORC1 by ablating the *Tsc1* gene during fasting can downregulate a subset of PPARα target genes, this effect is insufficient to prevent fasting-induced ketogenesis [113,114]. These contradictory findings highlight the complexity of metabolic regulation and the need for further investigation.

**Figure 5:**
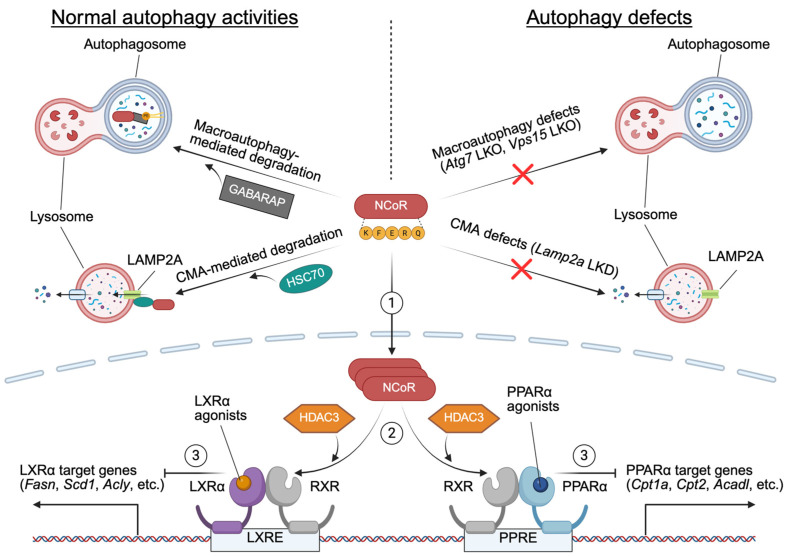
The role of autophagy in controlling nuclear receptor function via NCoR degradation. In healthy hepatocytes, the nuclear receptor corepressor NCoR is normally degraded through macroautophagy or CMA, involving interaction with proteins such as GABARAP (for macroautophagy) or HSC70 (for CMA), respectively. When either macroautophagy or CMA is impaired, NCoR is no longer efficiently degraded and instead accumulates in hepatocytes. This accumulated NCoR protein is translocated to the nucleus, where it acts as a corepressor for nuclear receptors LXRα and PPARα by recruiting HDAC3. As a result, the expression of target genes regulated by these nuclear receptors is suppressed, leading to disruption in various metabolic pathways. Arrows indicate activation, while bars indicate repression. CMA, chaperone-mediated autophagy; LAMP2A, lysosome-associated membrane glycoprotein 2 isoform A; HSC70, heat shock cognate 70; LKO, liver-specific knockout; LKD, liver-specific knockdown; HDAC3, histone deacetylase 3; LXRE, LXR response element.

Nevertheless, subsequent studies have reaffirmed the importance of NCoR-mediated nuclear receptor repression in autophagy-deficient conditions. *Alb-Cre*; *Atg7^F/F^* or *Alb-Cre*; *Vps15^F/F^* mice show increased accumulation of transcriptional repressors HDAC3, NCoR, or both, which suppress the activity of PPARα and LXRα. These nuclear receptors are critical for FAO, ketogenesis, and de novo lipogenesis. Fasting-induced autophagy is necessary for NCoR degradation via the interaction of GABARAP family proteins, resulting in PPARα activation and induction of its target genes. These findings highlight the critical role of hepatic autophagy in maintaining metabolic homeostasis during fasting by fine-tuning the levels of transcriptional repressors such as NCoR [7,8,12] (Figure 5).

Recent research has expanded our understanding of autophagy’s role in nuclear receptor regulation beyond macroautophagy. Choi et al. demonstrated that chaperone-mediated autophagy (CMA) also contributes to NCoR degradation by interacting with heat shock cognate 70 (HSC70). The accumulation of hepatic NCoR in aged mice or a genetic ablation of LAMP2A, a key component of CMA, results in PPARα inactivation and suppression of FAO [233]. This discovery links CMA to nuclear receptor regulation and expands the scope of autophagy’s impact on hepatic metabolism (Figure 5).

Taken together, these studies emphasize the multifaceted roles of autophagy in maintaining hepatic lipid homeostasis. By regulating the levels of transcriptional repressors through various degradation pathways, autophagy markedly affects the expression and/or activity of nuclear receptors, balancing lipogenesis, FAO, and ketogenesis. This intricate regulation has significant implications for understanding and potentially treating metabolic disease, age-related liver dysfunction, and other hepatic disorders associated with impaired autophagy or nuclear receptor signaling.

However, a distinct mechanism has been proposed regarding the attenuation of hepatic nuclear receptor activation in autophagy-deficient mouse models. This mechanism involves the p62-KEAP1-NRF2 axis: Genetic deletion of core-autophagy genes (e.g., *Atg7*, *Atg5*) in mice triggers marked accumulations of the autophagy receptor p62, which binds KEAP1, a component of Cullin-3-type ubiquitin ligase via the p-STGE motif in the KEAP1-interacting region (KIR). This interaction prevents NRF2 from undergoing ubiquitin-proteasome system (UPS)-mediated degradation, leading to NRF2 nuclear translocation. In the nucleus, NRF2 forms a heterodimer with small Maf proteins (e.g., MafF, MafG, and MafK) to bind and activate antioxidant response elements (AREs), inducing cytoprotective genes such as *Nqo1*, *Gstm1*, and *Cyp2a5* [234,235]. While this axis mitigates oxidative stress in autophagy-deficient tissues, it paradoxically drives the development of hepatomegaly, liver injury, and HCC in autophagy-deficient mouse models (e.g., *Mx1-Cre*; *Atg7^F/F^* mice). Global deletion of the *Sqstm1* gene encoding p62 or the *Nfe2l2* gene expressing NRF2 rescues these hepatic abnormalities, confirming their causative roles [219,231]. This suggests that the accumulation of p62 and subsequent NRF2 hyperactivation exemplify dual roles of autophagy: while essential for protein homeostasis, its deficiency disrupts transcriptional regulation, creating a toxic imbalance between cytoprotection and metabolic dysfunction. This interplay demonstrates that autophagy maintains a healthy liver by preventing pathogenic protein aggregation and uncontrolled antioxidant signaling. The p62-KEAP1-NRF2 axis thus emerges as a critical but double-edged mediator in autophagy-deficient livers, linking proteostasis failure to transcriptional dysregulation and disease progression.

**Figure 6:**
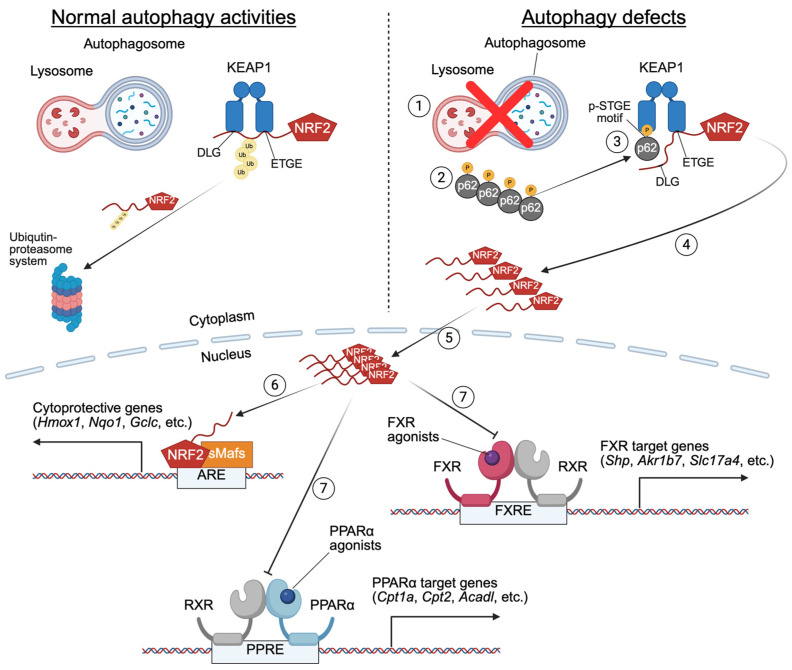
The role of autophagy in controlling nuclear receptor function via the p62-KEAP1-NRF2 axis. In healthy hepatocytes, the E3 ligase KEAP1 binds to the antioxidant transcription factor NRF2 promoting its polyubiquitination and subsequent degradation via the ubiquitin-proteasome system (UPS). When macroautophagy is impaired, p62 accumulates in the cytoplasm and binds to KEAP1 via its p-STGE motif. This interaction prevents NRF2 degradation, allowing it to translocate into the nucleus. There, NRF2 forms a heterodimer with small Marf (sMaf) proteins and activates cytoprotective gene expression by binding AREs. However, nuclear accumulation of NRF2 also suppresses the activity and expression of nuclear receptors FXR and PPARα, leading to downregulation of their target genes. As a result, key multiple metabolic pathways—including bile acid biosynthesis, FAO, and ketogenesis—are disrupted. Arrows indicate activation, while bars indicate repression.

In this perspective, persistent NRF2 activation in autophagy-deficient mice (e.g., hepatocyte-specific *Atg5* or *Atg7* knockouts) disturbs transcriptional programs executed by certain nuclear receptors, particularly for FXR and PPARα, critical regulators of bile acid and lipid metabolism. The study by Khambu et al. revealed an intricate interconnection between autophagy, NRF2, and FXR in regulating hepatic BA metabolism and cholestasis. *Atg5* or *Atg7*-deficient livers exhibit severe intracellular cholestasis, characterized by elevated serum BA levels, malformed bile canaliculi, and dysregulated expression of bile transporters. These phenotypes are linked to suppressed FXR activity [9]. Whole-body *Fxr* null (*Fxr^−/−^*) mice mirror some of these cholestatic features, as do loss-of-function mutations in the human *FXR*/*NR1H4* gene. The latter exhibits progressive familial intrahepatic cholestasis (PFIC), with FXR classified as the 5th type of PFIC, underscoring the essential role of FXR in BA homeostasis [9,236,237]. Intriguingly, liver-specific or intestine-specific *Fxr* knockout (*Alb-Cre*; *Fxr^F/F^* or *Villin-Cre*; *Fxr^F/F^*) mice do not replicate this severity, suggesting systemic FXR loss might be required for full cholestasis [129,238]. NRF2-mediated suppression of FXR activity is further evidenced by studies in liver-specific *Keap1* knockout (*Alb-Cre*; *Keap1^F/F^*) mice or control wild-type mice treated with the pharmacological NRF2 activator butylated hydroxyanisole (BHA), both showing NRF2 hyperactivation and subsequently reduced FXR target gene expression. Conversely, ablating the global or liver-specific *Nfe2l2* gene (*Nfe2l2^−/−^* or *Alb-Cre*; *Nfe2l2^F/F^*) or pharmacological activation of FXR restores its activity in autophagy-deficient models, confirming the antagonistic role of NRF2 in regulating FXR function [9,11]. A similar mechanism also applies to PPARα: NRF2 activation in *Alb-Cre*; *Atg7^F/F^* mice suppresses PPARα-driven FAO, exacerbating metabolic dysfunction [10] (Figure 6).

While NRF2 activation in autophagy-deficient conditions has been demonstrated to suppress nutrient-sensing nuclear receptors PPARα and FXR, the precise mechanisms remain unresolved [239]. However, previous studies provide us with some insights into the potential mechanisms including (1) coactivator competition, where NRF2 sequesters shared coactivators (e.g., CBP/p300), limiting their availability for PPARα and FXR [240,241,242]; (2) direct interactions between NRF2 and nuclear receptors (e.g., PPARα, FXR, etc.) or their heterodimer partner RXRs, blocking DNA binding [243]; (3) epigenetic remodeling, where NRF2 alters chromatin accessibility or histone modifications at nuclear receptor binding sites, resulting in dysregulated expression of target genes; (4) modulation of coregulators, where NRF2 regulates coactivator/corepressor expression critical for PPARα and FXR activity; and (5) redox modulation, as NRF2-driven shifts in cellular redox states may impair PPARα and FXR activity or their binding to target genes [244]. These mechanisms may act synergistically, but further validation is required.

To better understand the inhibitory effects of NRF2 on nuclear receptor signaling, it would be necessary to determine genome-wide analyses of cistromes, including transcription factors (e.g., PPARα, FXR, LXRα, RXRs, NRF2, etc.), coregulators (e.g., NCoR, SMRT, CBP/p300, etc.), and histone marks (e.g., H3K27ac, H3K27me3, etc.), in autophagy-deficient livers. Such studies could elucidate transcriptional conflicts and identify nodes for intervention. For instance, inhibitors targeting NRF2-coactivator interactions or agonists restoring PPARα and FXR activity might counteract cholestasis or other metabolic dysfunctions in diseases including PFIC5 or NAFLD. Understanding these mechanisms could unlock therapies for disorders where autophagy defects intersect with nuclear receptor dysfunction, bridging gaps between redox balance and metabolic regulation.

Although the p62–KEAP1–NRF2 axis is well established in autophagy-deficient hepatocytes, there is currently no literature investigating whether cytoplasmic p62 accumulation promotes NCoR nuclear translocation or vice versa. Therefore, the crosstalk between the KEAP1–NRF2 pathway and NCoR nuclear translocation remains unexplored. Nevertheless, this is a highly intriguing topic that warrants further investigation.

It is noteworthy that autophagy also contributes to hepatic differentiation and carcinogenesis by degrading Yes-associated protein (Yap), an effector of the Hippo pathway [245,246]. Previous studies have shown that activation of the p62-KEAP1-NRF2 axis is associated with the development of benign adenoma and hepatomegaly in autophagy-deficient mouse models [234,235]. It is interesting to note that in *Alb-Cre*; *Atg7^F/F^* mice, simultaneous deletion of the autophagy substrate Yap also restores several abnormalities (e.g., liver enlargement, fibrosis, progenitor cell proliferation, hepatocarcinogenesis) irrespective of the p62-KEAP1-NRF2 axis [247]. By degrading Yap, these results demonstrate autophagy as a gatekeeper of hepatic differentiation and tumor suppression, suggesting possible treatment options for liver cancer based on modulating autophagy or Yap activity. From this angle, it would be intriguing to investigate whether accumulated Yap proteins in autophagy-deficient cells also interfere with the activation of nutrient-sensing nuclear receptors PPARα and FXR.

## 7. Bidirectional Regulation Between Autophagy and Nutrient-Sensing Nuclear Receptors PPARα and FXR

The regulatory crosstalk between autophagy and the nutrient-sensing nuclear receptors PPARα and FXR operates through elaborate bidirectional mechanisms that ensure metabolic homeostasis in the liver. In the forward direction, PPARα and FXR exert opposing transcriptional output over autophagy: PPARα activation during fasting stimulates autophagy by upregulating autophagy-related genes, accelerating cellular component recycling and energy production, while FXR activation in the fed state suppresses autophagy via downregulation of these same genes. In the backward direction, autophagy provides critical feedback regulation of nuclear receptor activity through multiple mechanisms. Basal autophagy maintains the degradation of NCoR, whose accumulation in autophagy-deficient hepatocytes inhibits the transactivation of PPARα and FXR. Additionally, impaired autophagy leads to enhanced cytoplasmic interactions between p62 (Sequestosome 1) and the E3 ligase KEAP1, promoting nuclear translocation of NRF2, which subsequently interferes with PPARα and FXR transactivation through unclear mechanisms. This reciprocal regulation is functionally critical, as liver-specific deletion of core autophagy genes (e.g., *Atg5* or *Atg7*) remarkably blunts the expression of PPARα and FXR target genes involved in FAO, ketogenesis, and bile acid homeostasis, ultimately leading to metabolic dysfunction and intrahepatic cholestatic injury. This bidirectional relationship ensures that autophagy and nuclear receptor signaling are coordinately regulated to match the liver’s metabolic needs during different nutritional states.

## 8. Clinical Implications and Therapeutic Potential of Targeting the Interplay Between Autophagy and Nutrient-Sensing Nuclear Receptors PPARα and FXR

To translate mechanistic insights into effective therapies, it is essential to first address the clinical and translational implications of the PPARα–FXR–autophagy axis. A major limitation of current pharmacological strategies lies in the non-specific activation of nuclear receptors, which can lead to undesirable side effects such as hepatotoxicity, gastrointestinal disturbances, and metabolic imbalance. To overcome these challenges, tissue-specific drug delivery systems—such as liver-targeted nanocarriers for nuclear receptor agonists or liver-restricted ligands—should be developed to enhance efficacy while minimizing off-target effects. Furthermore, temporal regulation strategies that align treatment with circadian rhythms or metabolic states may help prevent receptor desensitization and preserve physiological balance. Importantly, autophagy not only influences the generation of endogenous ligands but also regulates the nuclear localization and activity of transcriptional corepressors such as NCoR and HDAC3. Thus, consideration of autophagic status is critical when designing precision therapies.

Building on this framework, restoring or modulating autophagic activity in conjunction with pharmacological manipulation of PPARα or FXR represents a promising therapeutic approach for rebalancing hepatic metabolism. These nutrient-sensing nuclear receptors interact closely with autophagic processes to regulate lipid oxidation, bile acid homeostasis, and energy utilization. When activated in a spatially and temporally controlled manner, these pathways can synergize to correct metabolic dysfunction and mitigate disease progression. This integrated strategy may hold strong potential for improving clinical outcomes in chronic liver disorders such as nonalcoholic fatty liver disease (NAFLD), nonalcoholic steatohepatitis (NASH), and primary biliary cholangitis (PBC). We believe these approaches will not only enhance therapeutic precision but also mitigate adverse effects, thereby improving the translational potential of PPARα and FXR-targeted interventions in metabolic liver diseases.

## 9. Conclusions

PPARα and FXR, two essential nutrient-sensing nuclear receptors, play critical roles in regulating hepatic energy homeostasis by coordinating lipid metabolism, bile acid homeostasis, and glucose production in fasting and feeding states. Their activities are tightly linked to autophagy, which maintains proper nuclear receptor signaling by controlling corepressor degradation, coactivator availability, and redox balance. Autophagy deficiency disrupts this intricate balance, leading to impaired activity of PPARα and FXR, metabolic dysfunction, and liver pathologies, such as steatosis and cholestasis.

The interplay between autophagy and nuclear receptor signaling is further complicated by NRF2 activation in autophagy-deficient conditions. NRF2 may compete with nuclear receptors for coactivators, interfere with DNA binding, or modulate redox-sensitive transcriptional programs, ultimately suppressing PPARα- and FXR-mediated metabolic pathways. While NRF2 activation provides cytoprotective effects against oxidative stress, its chronic hyperactivation in the absence of autophagy exacerbates liver dysfunction.

These findings underscore the critical role of autophagy in sustaining nuclear receptor function and maintaining metabolic homeostasis in the liver. Therapeutic strategies targeting the p62-KEAP1-NRF2 axis or enhancing PPARα/FXR activity could restore metabolic balance and mitigate liver pathogenesis associated with autophagy dysfunction. Future research should focus on elucidating the precise molecular mechanisms underlying NRF2-nuclear receptor crosstalk and exploring genome-wide transcriptional dynamics to identify new therapeutic targets. Understanding these complex interactions will pave the way for innovative treatments for liver diseases linked to autophagy impairment and nuclear receptor dysregulation.

## Figures and Tables

**Table 1:** Phenotypes of liver-specific knockout mice of *Atg* genes related to autophagy initiation.

Gene		Model	Phenotype	Reference
Autophagy initiation	*Ulk1/2*	*Alb-Cre*; *Ulk1^F/F^*; *Ulk2^−/−^*	Normal autophagy activity, mild hepatomegaly, protection from APAP-induced liver injury, unaltered hepatic TG/cholesterol levels	[208]
	*Fip200*	*Alb-Cre*; *Fip200^F/F^*	Severe hepatomegaly, enlarged hepatocytes, ubiquitin-positive aggregates, p62 accumulation, lower serum and hepatic TG levels (fasting/HFD), impaired LXRα activity, increased susceptibility to endotoxin-induced liver injury	[209]
	*Atg13*, *Atg17*, *Atg101*	No liver-specific knockout reported	N/A	N/A

Alb-Cre, expressing CRE recombinase under the control of albumin promoter; APAP, acetaminophen; TG, triglyceride; HFD, high-fat diets; LXRα, liver x receptor α.

**Table 2:** Phenotypes of liver-specific knockout mice of *Atg* genes related to vesicle nucleation.

Gene		Model	Phenotype	Reference
Vesicle nucleation	*Vps34*	*Alb-Cre*; *Vps34^F/F^*	Hepatomegaly, hepatic steatosis, decreased protein turnover, impaired AP production during fasting, blunted amino acid-mediated mTOR signaling	[210]
	*Vps15*	*Vps15^F/F^ injected with Adeno-Cre (IV)*	Liver enlargement, decreased autophagy activity, increased size and number of hepatocytes, p62 accumulation, vacuolization in hepatocytes, altered LC3 lipidation	[211]
		*Alb-Cre*; *Vps15^F/F^*	Mitochondrial depletion, impaired FAO/ketogenesis, and compromised PPARα activation due to NCoR and HDAC3 accumulation	[7]
	*Nrbf2*	*Nrfb2^−/−^*	Impaired ATG14L-linked VPS34 activity, decreased vesicle nucleation, enhanced ER stress-mediated cytotoxicity, focal liver necrosis, ductular reaction	[212]
	*Becn1*, *Atg14L*, *Ambra1*, *p115*	No liver-specific knockout reported	N/A	N/A

Alb-Cre, expressing CRE recombinase under the control of albumin promoter; AP, autophagosome; mTOR, mammalian/mechanistic target of rapamycin; Adeno-Cre, adenovirus expressing CRE recombinase; IV, intravenous injection; FAO, fatty acid oxidation; PPARα, peroxisome proliferator-activated receptor α; NCoR, nuclear receptor corepressor; HDAC3, histone deacetylase 3.

**Table 3:** Phenotypes of liver-specific knockout mice of *Atg* genes related to vesicle elongation.

Gene		Model	Phenotype	Reference
Vesicle elongation	*Atg3*	*Atg3* knockdown in hepatocytes	Lipid accumulation (CDHFD), enhanced fatty acid catabolism, elevated mitochondrial activity, SIRT1-mediated deacetylation, CPT1α-driven fatty acid transport to mitochondria	[216]
	*Atg5*	*CAG-Cre*; *Atg5^F/F^* (ubiquitous expression of CRE)	Mosaic deletion of *Atg5* in hepatocytes, accumulation of cytoplasmic ubiquitinated proteins and p62, benign liver adenomas, mitochondrial swelling, oxidative stress and DNA damage	[217,218]
		*Mx1-Cre*; *Atg5^F/F^* (inducible expression of CRE)	Inducible *Atg5* deletion (via pIpC injection), time-dependent progression of protein aggregation, initial diffuse ubiquitinated proteins, large inclusion bodies by day 16 post-injection, disrupted proteostasis precedes visible aggregate formation	[219]
		*Alb-Cre*; *Atg5^F/F^*	Liver pathologies (apoptosis, inflammation, fibrosis, HCC), reduced hepatic lipid accumulation during fasting, impaired ketogenesis, defective starvation-induced LD formation, pathologies attenuated by co-deletion of NRF2, persistent NRF2 activation disrupts fasting-induced lipid mobilization	[217,220,221,222]
	*Atg7*	*Mx1-Cre*; *Atg7^F/F^* (inducible expression of CRE)	Complete deletion of *Atg7* in liver and spleen; partial deletion of *Atg7* in kidney and heart, hepatomegaly, disorganized hepatic lobules, swollen/vacuolated hepatocytes with cell death, increased peroxisome biogenesis, elevated ubiquitin-positive aggregates, increased ALT, AST, and ALP levels indicating severe liver injury	[223]
		*Alb-Cre*; *Atg7^F/F^* (Singh et al.)	Elevated hepatic TG/cholesterol levels (fed and fasted), hepatomegaly, hepatic steatosis, reduced TG secretion due to impaired LD lipolysis, autophagy necessary for LD breakdown, TG release, and FAO	[224]
		*Alb-Cre*; *Atg7^F/F^* (Other studies)	Reduced TG levels during fasting or hepatectomy, decreased LD size and number; autophagy is essential for fasting-induced LD biogenesis	[12,225]
		*Alb-Cre*; *Atg7^F/F^* (Kim et al.)	Hepatomegaly, irregular hepatic lobules, decreased hepatic lipid levels and TG secretion during fasting, reduced gene expression involved in fatty acid synthesis, TG production, and FAO, improved glucose homeostasis via elevated hepatic FGF21 production through ATF4 activation, decreased BW, fat mass, and hepatic steatosis under chow or HFD, diminished fasting-induced ketogenesis due to impaired PPARα activity	[8,226]
		*ERt-Alb-Cre*; *Atg7^F/F^*	Severe liver damage induced by GalN/LPS treatment, increased apoptosis via caspase-8 activation and mitochondrial cell death pathway, autophagy protects against TNFα-mediated tissue damage by alleviating apoptotic signaling pathways	[227]
	*Atg2*, *Atg4*, *Atg8*, *Atg9*, *Atg10*, *Atg12*, *Atg16l*	No liver-specific knockout reported	N/A	N/A

CDHFD, choline-deficient high-fat-diets; SIRT1, sirtuin 1; CPT1α, carnitine palmitoyltransferase 1α; CAG-Cre, expressing CRE recombinase under the control of CAG promoter; Mx1-Cre, expressing CRE recombinase under the control of Mx1 promoter; pIpC, polyinosinic-polycytidylic acid; Alb-Cre, expressing CRE recombinase under the control of albumin promoter; HCC, hepatocellular carcinoma; LD, lipid droplet; NRF2, nuclear factor erythroid 2-related factor 2; ALT, alanine transaminase; AST, aspartate transferase; ALP, alkaline phosphatase; TG, triglyceride; FAO, fatty acid oxidation; FGF21, fibroblast growth factor 21; ATF4, activating transcription factor 4; BW, body weight; HFD, high-fat diets; PPARα, peroxisome proliferator-activated receptor α; ERt-Alb-Cre, expressing CRE recombinase fused with estrogen receptor ligand-binding domain under the control of albumin promoter; GalN, D-galactosamine; LPD, lipopolysaccharide.

**Table 4:** Phenotypes of liver-specific knockout mice of *Atg* genes related to the docking and fusion of AP-lysosome.

Gene		Model	Phenotype	Reference
Docking and fusion of AP-lysosome	*Pacer*	*Alb-Cre*; *Pacer^F/F^*	Impaired autophagy and metabolic fluxes, TG and glycogen accumulation, reduced ketogenesis, early-onset fibrosis (increased collagen deposition) and liver injury, no hepatomegaly and HCC	[229]
	*Rubcn*	*Alb-Cre*; *Rubcn^F/F^*	Enhanced autophagy, protection against HFD-induced hepatic steatosis, liver damage and ER stress, no abnormalities on NCD	[230]
	*Becn1*, *Uvrag*, *Stx17*	No liver-specific knockout reported	N/A	N/A

AP, autophagosome; Alb-Cre, expressing CRE recombinase under the control of albumin promoter; TG, triglyceride; HCC, hepatocellular carcinoma; HFD, high-fat diets; ER stress, endoplasmic reticulum stress; NCD, normal chow diets.

## Data Availability

No new data were created and analyzed in this study. Data sharing is not applicable to this article.
